# Impact of Diabetes on Excessive Cardiovascular Risk: Matched Analysis Based on the European Health Interview Survey

**DOI:** 10.3390/medicina60121928

**Published:** 2024-11-23

**Authors:** Jenifer Pataki, Gergő József Szőllősi

**Affiliations:** 1 Department of Integrative Health Sciences, Faculty of Health Sciences, University of Debrecen, 4032 Debrecen, Hungary; pataki.jenifer@etk.unideb.hu; 2 Coordination Center of Research in Social Sciences, Faculty of Economics and Business, University of Debrecen, 4032 Debrecen, Hungary

**Keywords:** diabetes, cardiovascular disease, propensity score matching, excess risk, European health interview survey

## Abstract

*Background and Objectives*: Diabetes represents a major public health challenge due to its strong link to cardiovascular risk, therefore the aim was to explore the excessive cardiovascular risk attributed to diabetes. *Materials and Methods*: This cross-sectional study was based on data from the European Health Interview Surveys in Hungary. Propensity score matching was used to control confounding factors including age, gender, education, marital status, income, health status, obesity, smoking, place of residence, and physical activity. *Results*: A total of 15,874 individuals were analyzed, of whom 1447 (9.12%) reported having diabetes. Furthermore, diabetes was significantly associated with higher prevalence rates of hypertension (by 23.4%), arrhythmia (by 3.85%), heart attack (by 3.42%), and coronary artery disease (by 6.92%) after adjusting for potential confounders. *Conclusions*: These findings highlight the importance of managing cardiovascular risk factors in individuals with diabetes.

## 1. Introduction

With its prevalence rising at an alarming rate, diabetes has become one of the most imperative and urgent global health issues [1,2]. Over 422 million adults worldwide currently have diabetes; this number is expected to rise in the next decades as aging populations and changing lifestyles add to the disease’s burden [1,3,4]. This long-term metabolic condition, marked by poor glucose control, has a substantial negative impact on quality of life as well as health care systems [5]. The most serious consequences of diabetes are cardiovascular diseases (CVDs), which include coronary artery disease, hypertension, stroke, arrhythmia, and heart attacks, that contribute to diabetes related morbidity and mortality [2,5].

Individuals with diabetes have a two- to fourfold higher risk of developing CVDs than those without the condition [6]. This is due in part to the negative effects of prolonged high blood sugar levels on blood vessels and the cardiovascular system. Diabetes hastens the progression of atherosclerosis, thickening arterial walls and resulting in heart attacks and strokes [7]. These cardiovascular complications significantly reduce diabetic patients’ life expectancy and are the leading cause of death in this group [8].

Diabetes prevalence in Hungary has steadily increased over the last few decades, mirroring global trends [9]. Consequently, diabetes and cardiovascular diseases pose a significant public health burden in the country, since CVDs are still the leading cause of death in Hungary [10]. Because of the increasing rate of people being diagnosed with diabetes, the overlap between these two conditions is becoming more concerning. Understanding how diabetes affects cardiovascular health in this context is essential for developing and implementing adequate public health strategies to reduce the prevalence and impact of these co-occurring conditions [11,12,13].

Although the relationship between diabetes and cardiovascular diseases could be considered a well-known and researched public health topic, it still remains essential to identify the potential confounding variables, such as age, educational attainment, and lifestyle behaviors, for this association since the understanding of these factors is crucial to accurately assess the independent impact of diabetes on cardiovascular risk [14,15].

The main aim of this study was to investigate the relationship between diabetes and various cardiovascular comorbidities, including hypertension, stroke, arrhythmia, heart attack, and coronary artery disease for quantifying the excess risk attributable to diabetes by using propensity score matching to control for potential confounders.

## 2. Materials and Methods

This cross-sectional study was based on representative data from the European Health Interview Survey (EHIS) executed in Hungary, which provides comprehensive reliable health-related data on adults [16]. The survey included detailed information on individuals’ health status, medical history, lifestyle, and socio-demographic characteristics. The study population included individuals aged 18 or above from the EHIS dataset who reported information regarding diabetes status and cardiovascular conditions. Individuals with missing or incomplete data on key variables were excluded from the confounder-adjusted analysis.

The description of study variables can be seen in Table 1. The main grouping variable of potential interest was the self-assessed diabetes status (Yes/No), and the primary outcomes of this study were the five cardiovascular conditions: hypertension (Yes/No), stroke (Yes/No), arrhythmia (Yes/No), heart attack (Yes/No), and coronary artery disease (Yes/No); all based on self-report. The multiple-multivariate propensity matching score analyses were adjusted for several confounding including the respondents’ age group (18–34/35–64/65–X), gender (Male/Female), educational attainment (Primary/Secondary/Tertiary), marital status (Partnered/Not Partnered), type of residence (Urban/Rural), self-perceived household income (Good/Bad), self-perceived health status (Good/Bad), obesity status (Yes/No), smoking status (Yes/No), respondents’ region of residence (Central Hungary/Southern Great Plain/Southern Transdanubia/Central Transdanubia/Western Transdanubia/Northern Great Plain/Northern Hungary), and the level of physical activity (Inactive/Minimally Active/Active).

Descriptive statistics were obtained with Fisher’s exact tests per diabetes strata. To reduce the bias resulting from potential confounding factors as well as to estimate the effect of diabetes on cardiovascular outcomes, propensity score matching was executed. The propensity scores for each individual were estimated using logistic regression models which included all the confounding variables listed above. This was followed by the nearest-neighbor matching algorithm with a caliper width of 0.05 to match individuals with diabetes to those without diabetes, based on their propensity scores. To verify covariate balance after propensity score matching, standardized mean differences (SMDs) were calculated for each covariate, with values below 0.1 indicating acceptable balance and values under 0.05 indicating excellent balance. Furthermore, overall balance assessment was performed using metrics such as mean bias, median bias, pseudo R-squared, and the B and R statistics, all of which were within acceptable ranges. The average treatment effect with t-statistics on the treated (ATT) was estimated to assess the excess risk of cardiovascular outcomes attributable to diabetes. Differences in proportions between the diabetes and matched control groups were calculated for each outcome. The statistical analysis was performed using Stata Statistical Software (version 13.0, Stata Corp, College Station, TX, USA).

## 3. Results

The total sample size for the analysis was 15,874 with 1447 (9.12%) reporting diabetes and 14,427 (90.88%) not having diabetes.

A significant difference was found regarding age group and diabetes, with diabetes prevalence increasing from 1.43% in the youngest age group (aged between 18–34) to 19.22% in the oldest (aged 65 or above) age group significantly (*p* < 0.001). Among men, 9.29% had diabetes, compared to 8.97% of women who had diabetes, but there was no statistically (*p* = 0.489) proven difference regarding gender and diabetes status, indicating that gender does not appear to have a significant effect in diabetes prevalence in this sample based on the univariate analysis. Educational attainment was also significantly (*p* < 0.001) linked to diabetes status, since the diabetes prevalence was 13.63% among diabetic people with primary educational level, compared to 6.52% among those with the highest educational level. Marital status showed a significant (*p* < 0.001) difference across diabetic strata, with diabetes prevalence being higher among partnered or married individuals (10.02%) compared to non-partnered diabetic respondents (8.04%). Diabetes was significantly (*p* < 0.001) more frequent (11.15%) among people with worse self-perceived income status when compared with diabetic individuals with good self-perceived income (7.62%). People with poor self-perceived health status had significantly (*p* < 0.001) higher prevalence of diabetes of 24.18% compared to 6.88% of those with good self-assessed health status. Obesity was a significant (*p* < 0.001) factor towards diabetes, with 12.89% of overweight or obese individuals having diabetes, versus 3.93% among non-obese individuals. Physical activity showed significant (*p* < 0.001) inverse association regarding diabetes, with the lowest physical activity group having the highest prevalence of 14.63% compared to 6.00% in the most active group. Smoking status showed a lower prevalence of diabetes among smokers (6.14%) compared to non-smokers (10.28%). Regional differences in diabetes prevalence were observed in Hungary, but no significant (*p* = 0.814) difference was found based on residence type.

Based on the univariate descriptive statistics, diabetes was significantly more frequent among individuals with cardiovascular conditions (Table 2). For hypertension, only 3.34% of those without the condition had diabetes, while this increased to 19.95% among those with hypertension, showing a significant (*p* < 0.001) difference. A similar pattern could be observed for stroke, since 8.73% of individuals without a history of stroke had diabetes, whereas 23.49% of those who have had a stroke are diabetic, highlighting a significant (*p* < 0.001) contrast. In terms of arrhythmia, 7.74% of respondents without the condition had diabetes, but this rose to 19.04% among those with arrhythmia significantly (*p* < 0.001). This difference could be observed regarding heart attacks because 8.52% of those without a history of heart attack considered themselves diabetic, compared to 26.72% of those who have had at least one such cardiovascular event. Finally, for coronary artery disease, 7.98% of individuals without this condition suffered from diabetes while 27.05% of those with the disease considered themselves diabetic.

Based on the multiple propensity matched analysis results, 76.10% [73.80–78.26%] of individuals with diabetes had hypertension, compared to 30.76% [30.00–31.52%] of those without diabetes, showing a significant difference of 45.34% (Table 3). After matching for confounding factors, the prevalence of hypertension remained 76.10% [73.80–78.26%] in the diabetes group, while it decreased to 52.70% [50.02–55.24%] in the matched control group, which means that there was a substantial difference of 23.40%. This might suggest that diabetes significantly increased the likelihood of hypertension. Before matching, 6.83% [5.44–8.07%] of people with diabetes have experienced a stroke, compared to 2.19% [1.95–2.43%] of those without diabetes, showing a slight difference of 4.63%. However, after matching, the stroke rate among those with diabetes was approximately 6.83% [5.44–8.07%], nearly identical to the 6.90% [5.57–8.22%] observed in the control group, indicating no significant difference. This suggests that the apparent association between diabetes and stroke in the unmatched comparison is likely due to confounding factors. Regarding arrhythmia, before matching, 25.59% [23.30–27.87%] of individuals with diabetes had this cardiovascular problem, compared to 10.86% [10.34–11.37%] without diabetes, revealing a difference of 14.73%. After the matching process, the rate of arrhythmia in the diabetes group remained 25.59% [23.30–27.87%], while in the control group it was equal to 21.74% [19.51–23.82%], resulting in a relatively small but significant difference of 3.85%. This indicates a modest association between diabetes and an increased risk of arrhythmia. Prior to matching, 9.61% [8.00–11.07%] of people with diabetes have had a heart attack, compared to 2.60% [2.33–2.86%] of those without diabetes, a difference of 7.00%. After matching, the heart attack rate in the diabetes group remained 9.61% [8.00–11.07%], while it was 6.19% [4.87–7.37%] in the control group, showing a significant difference of 3.42%. This might suggest that diabetes continues to be a significant risk factor for heart attacks even after accounting for other potentially confounding variables. Among people with diabetes, 17.48% [15.49–19.46%] suffered from a coronary artery disease, compared to 4.75% [4.40–5.10%] of those without, revealing a difference of 12.72%. After adjusting for other confounding factors, this rate remained 17.48% [15.49–19.46%] in the diabetes group, while it increased to 10.56% [8.95–12.16%] in the control group, resulting in a small significant difference of 6.92%. This shows that diabetes might raise the risk of coronary artery disease as well.

## 4. Discussion

This study described the association between diabetes and cardiovascular comorbidities based on a large, representative sample from the European Health Interview Surveys conducted in Hungary. The results quantified the effect of diabetes on several cardiovascular diseases, including hypertension, arrhythmia, heart attack, and coronary artery disease in confounder adjusted models to provide more accurate estimates of the independent impact of diabetes on cardiovascular risk. After adjusting for key confounders, diabetes remained a significant risk factor for most cardiovascular conditions, suggesting that diabetes might independently contribute to cardiovascular disease risk.

Hypertension, in particular, showed a markedly higher prevalence in the diabetic group even after matching, with 76.10% of diabetic individuals affected. This aligns with existing research, which has demonstrated that diabetes and hypertension often co-occur, substantially increasing the risk of other adverse cardiovascular events [17,18,19].

While diabetes was significantly associated with stroke in the unadjusted analysis, this association was no longer significant after propensity score matching, which may suggest the influence of residual confounding. Therefore, the relationship between diabetes and stroke may be confounded by other factors such as age or comorbidities like hypertension, which were not included in the current research [20,21,22]. Additionally, factors such as diet, medication adherence, and other lifestyle behaviors are important contributors to stroke risk and may influence the association between diabetes and stroke [23,24]. The finding that stroke risk did not remain significantly elevated among diabetic individuals after adjustment highlights the importance of considering the complex interplay of risk factors that contribute to cardiovascular outcomes in diabetic populations. The mixed findings on diabetes and stroke in our study highlights the importance of further research to better understand this relationship. The results of this study also emphasize the important role of lifestyle factors in mediating the relationship between diabetes and cardiovascular diseases [25,26].

In terms of cardiovascular comorbidities, diabetes increased the risk of heart attack, arrhythmia, and coronary artery disease even after adjusting for confounders, which aligns with the current scientific literature [15,27,28,29]. Therefore, moderate associations observed between diabetes and cardiovascular outcomes which may reflect a relatively complex interplay of biological, lifestyle, and behavioral factors [30,31,32,33]. These findings align with existing evidence that diabetes promotes atherosclerosis and inflammation, accelerating cardiovascular disease progression [7,34]. Furthermore, hyperglycemia in diabetes can also lead to endothelial dysfunction, reducing blood vessel elasticity, and increasing susceptibility to hypertension and other cardiovascular complications [35,36]. Additionally, diabetes-related metabolic changes, such as dyslipidemia and insulin resistance, contribute to arterial plaque formation, heightening the risk of events like stroke and heart attack [37]. These mechanisms, along with lifestyle factors like physical inactivity and dietary habits highlight the importance of targeted interventions to mitigate these risks including the use of sodium-glucose co-transporter-2 (SGLT2) inhibitors, a novel therapeutic choice in diabetes management that provides glycemic control through renal glucose excretion while offering significant cardiovascular benefits [38,39,40]. These might include reductions in blood pressure, weight, and inflammation, as well as renal protective effects. Hence, studies show SGLT2 inhibitors could reduce major adverse cardiovascular events and cardiovascular mortality in high-risk diabetic patients. These benefits align with our findings on the excessive cardiovascular risk in diabetes, particularly for hypertension and coronary artery disease. Hence, integrating SGLT2 inhibitors into standard diabetes care could significantly reduce cardiovascular risk [38,39,40]. Future research should explore their impact in diverse populations to guide personalized treatment strategies.

The findings emphasize the critical role of diabetes as a major risk factor for cardiovascular diseases and highlight the importance of comprehensive cardiovascular risk management, including blood glucose control, for reducing the burden of these life-threatening conditions in diabetic populations [41].

## 5. Limitations

Despite the valuable relationship described between diabetes and cardiovascular diseases by this study, several limitations must be noted. First, the cross-sectional design limits the ability to draw clear causal inferences about the relationship between diabetes and cardiovascular disease, even so longitudinal studies are required to confirm these findings and explore the temporal relationship between diabetes onset and cardiovascular outcomes.

Second, the reliance on self-reported data for both diabetes and cardiovascular conditions introduces the potential for recall bias or misclassification bias. Hence, underestimate or overestimate of the true prevalence of these conditions might occur, which could affect the strength of the observed associations. Furthermore, the analysis is limited by the exclusion of certain variables, such as dietary habits and medication adherence, due to data constraints within the EHIS dataset. In addition, age was treated as a categorical variable rather than a continuous one, which may have resulted in a loss of detailed information and subtle age-related variations that could influence cardiovascular risk. Even so, factors could act as unmeasured confounders potentially influencing cardiovascular risk; therefore, cardiovascular risk might not fully be described by the variables included in our model. Hence, while propensity score matching was used to adjust for confounding factors, residual confounding may still exist due to unmeasured variables.

## 6. Conclusions

In conclusion, this study reaffirms the relatively strong association between diabetes and cardiovascular comorbidities, even after adjusting for confounders, which means that diabetes is an imperative and independent risk factor for cardiovascular diseases. The findings highlight the need for targeted interventions to reduce cardiovascular risk in diabetic populations. Consequently, it could be essential for healthcare systems to adopt integrated care models that address both conditions simultaneously in order to mitigate the global health burden posed by diabetes and its cardiovascular complications

## Figures and Tables

**Table 1:** Description of study variables.

Variable Type	Variable	Categories/Levels
Grouping variable	Self-assessed diabetes status	Yes/No
Primary outcomes	Cardiovascular conditions	Hypertension (No/Yes) Stroke (No/Yes) Arrhythmia (No/Yes) Heart attack (No/Yes) Coronary artery disease (No/Yes)
Covariates	Age group	18–34/35–64/65+
	Gender	Male/Female
	Educational attainment	Primary/Secondary/Tertiary
	Marital status	Partnered/Not Partnered
	Type of residence	Urban/Rural
	Self-perceived household income	Good/Bad
	Self-perceived health status	Good/Bad
	Obesity status	No/Yes
	Smoking status	No/Yes
	Physical activity	Inactive/Minimally Active/Active
	Region	7 Regions

**Table 2:** Distribution of cardiovascular conditions by diabetes status (with row and column percentages).

Cardiovascular Diseases		Self-Assessed Diabetes Status		*p*-Value
		No	Yes	
Hypertension	No	9983 (96.66%) (69.33%)	345 (3.34%) (23.86%)	<0.001
	Yes	4417 (80.05%) (30.67%)	1101 (19.95%) (76.14%)	
Stroke	No	14,095 (91.27%) (97.81%)	1349 (8.73%) (93.29%)	<0.001
	Yes	316 (76.51%) (2.19%)	97 (23.49%) (6.71%)	
Arrythmia	No	12,830 (92.26%) (89.18%)	1077 (7.74%) (74.64%)	<0.001
	Yes	1556 (80.96%) (10.82%)	366 (19.04%) (25.36%)	
Heart attack	No	14,047 (91.48%) (97.41%)	1309 (8.52%) (90.59%)	<0.001
	Yes	373 (73.28%) 2.59%	136 (26.72%) (9.41%)	
Coronary artery disease	No	13,729 (92.02%) (95.30%)	1190 (7.98%) (82.58%)	<0.001
	Yes	677 (72.95%) (4.70%)	251 (27.05%) (17.42%)	

**Table 3:** Confounder-adjusted analysis: unmatched and propensity score matched comparison of cardiovascular disease prevalence in diabetic and non-diabetic individuals (with 95% confidence intervals). Significant findings are marked with “*” based on the T-statistics.

Cardiovascular Diseases	Unmatched Diabetic Prevalence (%)	Unmatched Non-Diabetic Prevalence (%)	Unmatched Difference (%)	Matched Diabetic Prevalence (%)	Matched Control Prevalence (%)	Matched Difference (%)
Hypertension	76.10 [73.80–78.26]	30.76 [30.00–31.52]	45.34 *	76.10 [73.80–78.26]	52.70 [50.02–55.24]	23.4 *
Stroke	6.83 [5.44–8.07]	2.19 [1.95–2.43]	4.63 *	6.83 [5.44–8.07]	6.90 [5.57–8.22]	−0.07
Arrhythmia	25.59 [23.30–27.87]	10.86 [10.34–11.37]	14.73 *	25.59 [23.30–27.87]	21.74 [19.51–23.82]	3.85 *
Heart attack	9.61 [8.00–11.07]	2.60 [2.33–2.86]	7.00 *	9.61 [8.00–11.07]	6.19 [4.87–7.37]	3.42 *
Coronary artery disease	17.48 [15.49–19.46]	4.75 [4.40–5.10]	12.72 *	17.48 [15.49–19.46]	10.56 [8.95–12.16]	6.92 *

## Data Availability

Data are available from the corresponding author upon a reasonable request.
